# The Use of a 27-Gauge Cannula in Aesthetic Medicine

**DOI:** 10.1093/asjof/ojac018

**Published:** 2022-03-24

**Authors:** Robyn Siperstein

In 2019, two publications recommended larger gauge cannulae for safer dermal filler treatments based on the decreased ability of larger cannulae to penetrate cadaver vessels.^1,2^ While they concluded that 25-gauge or larger cannulae are safer than 27-gauge cannulae,^1^ one noted several cases of vascular occlusion and even blindness with a 23- and 25-gauge cannula,^1,3-5^ leading one to hypothesize that there may be other factors that play a role in arterial penetration beyond the gauge of the cannula. The first study by Pavicic et al was a groundbreaking landmark study because it analyzed the arterial force of needles compared with cannulae, and there was minimal difference in the arterial penetration force with a select 27-gauge needle (0.70 N) when compared with a select 27-gauge cannula (0.78 N), questioning both the safety and utility of a 27-gauge cannula.^1^ However, like all studies, there were several limitations, most importantly the use of varying brands across both needles and cannulae creating a confounding variable as the penetration force of different brands of the same gauge cannula can vary significantly (0.8 to 2.2 N for 25-gauge cannulae) depending on the tapering, elastic modulus, and coating.^6^

**Figure 1.**
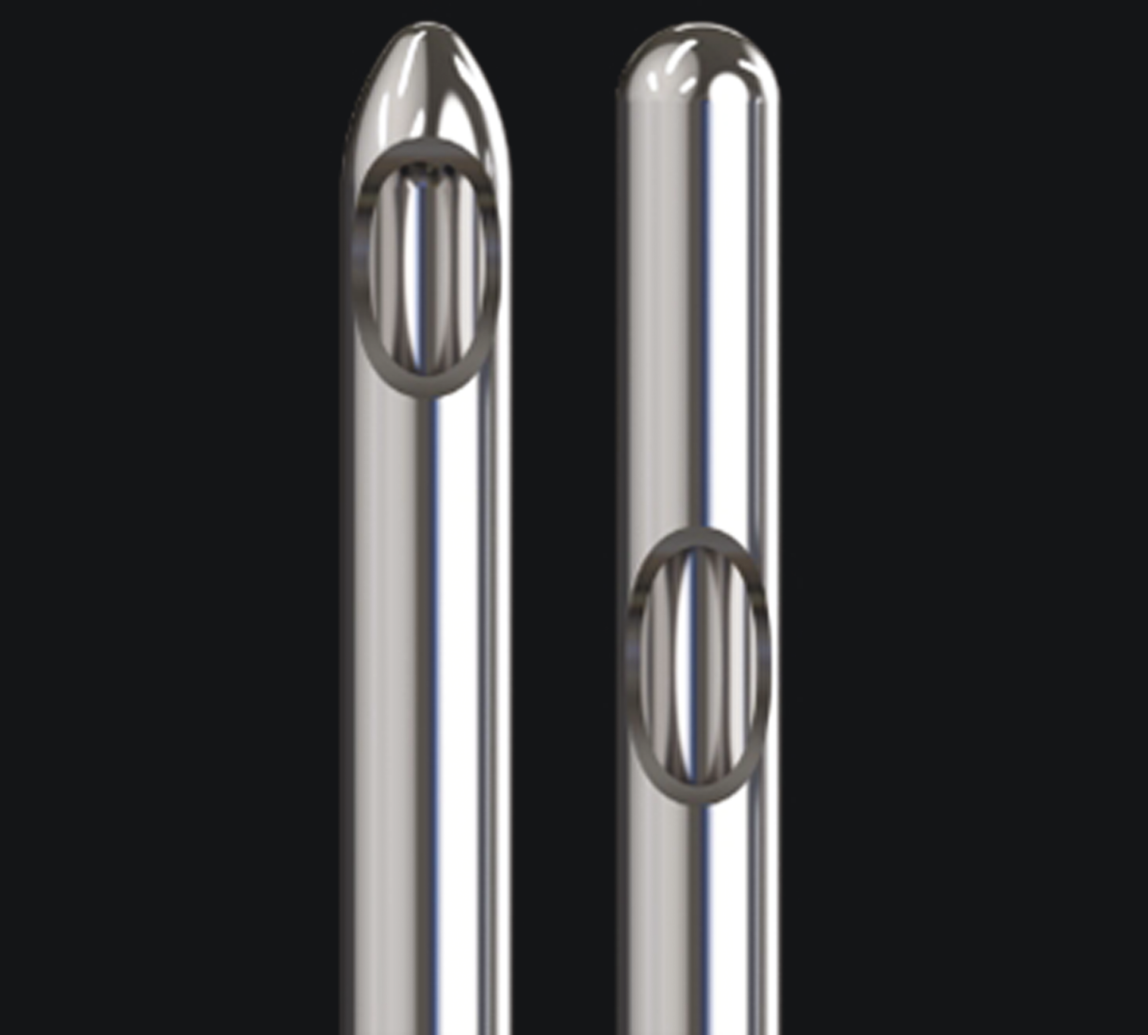
Cannula with a tapered end on the left (TSK Steriglide) adjacent to a cannula with a blunt end (TSK CSH) on the right. Image courtesy of TSK (Vancouver, BC, Canada).

In fact, in an animal study, one brand with a tapered head had a significantly lower arterial penetration force among 9 different brand cannulae despite all being the same gauge. The study also found an almost 2-fold difference in the elasticity of different brand cannulae with the same gauge.^6^ An image of 2 cannulae with the same gauge manufactured by the same company is shown in Figure 1.^7^ On the website, it describes the cannula on the left as having a “dome-shaped tip” which “creates less resistance than a traditional cannula tip” which “reduces the force” ^7^ needed through the tissue. However, one could hypothesize that, if there is less force through tissue, it is also possible there is less force to penetrate a vessel as seen in animal studies with this cannula type.^6^ In my opinion, the “resistance” we feel when encountering fibrotic tissue and ligaments with a blunt cannula that makes it more difficult to use is the same resistance we would feel with a vessel that allows us to redirect to increase safety.

It is important to briefly mention in any discussion of literature citing cadaveric anatomy that there is a marked difference to in vivo anatomy. The absence of living vascular dynamics affects vessel diameters and opposing forces, often with different measurable caliber of blood vessels in cadaveric specimens than in the living.^8^ Moreover, the surrounding environmental condition is different in a dissected cadaver as discussed further below, and cadavers tend to be older than the average age of those obtaining cosmetic fillers, with either more fragile or sclerotic tissues. In fact, in the 2019 study by Pavicic et al mentioned above, the average age of the cadavers was 74.5, and while there were only 4 cadavers limiting the study findings, there was a statistically significant correlation showing increasing age required decreasing force to penetrate an artery.^1^ Finally, while the cadavers in the 2 studies mentioned above were freshly frozen, some cadavers undergo a fixation process, where blood vessels have been shown to become more fragile due to changes in the biomechanical properties from protein cross-linking, which results in blood clotting and stiff, rigid, brittle vessels.^9^ Consequently, cadaveric anatomy should be interpreted with consideration of all postmortem changes, and the demographics of the cadavers when compared with the average patient undergoing cosmetic injectable treatments needs to be considered.

Since 2019, many injectors have switched over to larger gauge cannulae based on the literature. However, when injecting small delicate areas, such as the infraorbital hollow (IOH), the author continues to use a blunt-end, low elastic modulus (high elasticity), 27-gauge cannula (pictured to the right in Figure 1), as the author can feel resistance and redirect with that select 27-gauge cannula but not with the same gauge needle. Utilizing this 27-gauge cannula (CSH, TSK Laboratory; Vancouver, BC, Canada) and the author’s technique, most patients report decreased pain, bruising, and swelling when compared with a needle of the same gauge, which was also described in another study utilizing a 27-gauge cannula.^10^ In addition, after treating more than 1600 infraorbital hollows, there have been no reports of vascular occlusion and very limited reports of severe bruising despite having an older patient population (majority of patients above 50 years) and a significant number of patients on blood thinners. This is likely due to (1) the specific type of 27-gauge cannula which allows the author to feel resistance and redirect; (2) the minimal force used during advancement of the cannula; (3) injecting only retrograde while moving the cannula with minimal force on the plunger; (4) withdrawing the cannula most of the distance before wider re-direction to ensure that increase torque is not placed on fixed vessels; and (5) underlying vascular anatomy. While colleagues who use 27-gauge needles report that 5% to 50% of their IOH patients experience severe bruising or a “black eye,” the author’s rate remains around 1% when using a 27-gauge blunt-end cannula (TSK, CSH) and the assistance of a vein finder (AccuVein Inc; Medford, NY) for the entry port.

An additional consideration when comparing 2 gauges of identical cannulae is that while a larger gauge has increased arterial penetration force,^1,2^ it also would have increased tissue penetration force and less flexibility. This increased force to maneuver, especially through ligaments near vessels, is missed in cadaver studies. This effect could completely negate or dampen the increased safety of increasing arterial penetration force with larger cannulae because an increased force with the larger cannula may be required to pass through an area before arriving at a vessel in an adjacent plane. 

Finally, the same force on the plunger with a larger gauge cannula will deposit a larger amount of filler, which is more likely to cause a serious vessel blockage and possible blindness.^11^ While there is an inability to canalize vessels smaller than the diameter of the cannula being used, the angular artery in the infraorbital area measures approximately 0.9 mm,^12^ and therefore only an 18-gauge cannula would be larger (20-gauge cannulae measure 0.8 mm), which is not practical to use in the delicate IOH area. The author believes that cannula characteristics (blunt-end, elastic cannulae) and injection techniques (low force and redirecting with resistance) could be more important factors in decreasing the rate of vascular occlusions and bruising when compared with simply increasing the gauge of the cannula. Therefore, the author proposes that with the correct technique, a blunt-end, flexible 27-gauge cannula could still be an optimal tool in small, sensitive, delicate areas.

A future cadaveric study of human arterial penetration forces with the same gauge cannulae among different brands, as well as different gauges of the same brands, will help shed light on the effect of different cannula characteristics. Additionally, a prospective clinical trial detailing the rates of side effects with different gauge and brand cannulae will help explore the difference that the tapering, length, elastic modulus, gauge, coating, size of opening, and bevel of cannulae can make in clinical practice.
